# The use of spatially explicit genetic variation data from four deep-sea sponges to inform the protection of Vulnerable Marine Ecosystems

**DOI:** 10.1038/s41598-019-41877-9

**Published:** 2019-04-02

**Authors:** Cong Zeng, Malcolm R. Clark, Ashley A. Rowden, Michelle Kelly, Jonathan P. A. Gardner

**Affiliations:** 1 College of Animal Science and Technology, Hunan Agricultural University, Changsha, China; 2 School of Biological Sciences, Victoria University of Wellington, Wellington, 6140 New Zealand; 3 Coasts and Oceans National Centre, National Institute for Water and Atmospheric Research, Private Bag, 14901 Kilbirnie, Wellington New Zealand; 4 Coasts and Oceans National Centre, National Institute of Water and Atmospheric Research, Auckland, 1010 New Zealand

## Abstract

The United Nations General Assembly has called for greater protection of the world’s deep-sea species and of features such as Vulnerable Marine Ecosystems (VMEs). Sponges are important components of VMEs and information about their spatially explicit genetic diversity can inform management decisions concerning the placement of protected areas. We employed a spatially explicit hierarchical testing framework to examine genetic variation amongst archived samples of four deep-sea sponges in the New Zealand region. For *Poecillastra laminaris* Sollas 1886, significant mitochondrial (*COI*, *Cytb*) and nuclear DNA (microsatellite) genetic differences were observed between provinces, amongst north-central-south regions and amongst geomorphic features. For *Penares* sp. no significant structure was detected (*COI*, *12S*) across the same areas. For both *Neoaulaxinia persicum* Kelly, 2007 (*COI*, *12S*) and *Pleroma menoui* Lévi & Lévi 1983 (*COI*) there was no evidence of genetic differentiation within their northern only regional distributions. Of 10 separate species-by-marker tests for isolation-by-distance and isolation-by-depth, only the isolation-by-depth test for *N*. *persicum* for *COI* was significant. The use of archived samples highlights how historical material may be used to support national and international management decisions. The results are discussed in the broader context of existing marine protected areas, and possible future design of spatial management measures for protecting VMEs in the New Zealand region.

## Introduction

Larval dispersal and genetic connectivity are poorly understood in the deep sea, despite their importance in shaping patterns of biodiversity and biogeography, and their ability to provide insights to help guide the protection of rare and endangered species^1^. Analysis of spatially explicit patterns of genetic structure for multiple taxa can be used to evaluate the suitability of the location, size and spacing of marine protected areas (MPAs) and the establishment of MPA networks in the sea^2^. However, the identification of patterns of connectivity (gene flow), of regional genetic structure, and of genetic diversity hotspots is challenging in the deep sea because the high cost and logistical constraints of sampling make it difficult to acquire specimens at large spatial scales, and across a wide range of depths^1^. Given ongoing anthropogenic threats to the deep sea that include fishing, dumping, oil and gas exploration, and potential deep-sea mining^3^, there is an urgent need to better understand patterns of population genetic diversity and differentiation amongst vulnerable marine ecosystems (VMEs) so that appropriate protection mechanisms can be put in place^1,4^.

The relatively recent concept of VMEs has been advanced to help identify and thereby protect deep-sea species and features. VMEs are “assemblages of marine benthic organisms which are susceptible to anthropogenic disturbance, especially that arising from the impact of fishing gear used in bottom fishing”^5^. VME protection is called for by the United Nations General Assembly (UNGA), and such protection is the responsibility of bodies that manage resource use in areas beyond national jurisdiction (ABNJ) (e.g., Regional Fisheries Management Organisations, RFMOs)^6^. VMEs are typically characterised by taxa that are fragile and susceptible to damage (in particular from fishing gear), have high longevity and slow growth rates, and have a limited ability (or complete inability) to recover from disturbance^5^. VMEs are often associated with seamounts that are sites of intense fishing activity because some fish species aggregate around seamounts for breeding or feeding^7^. VME indicator taxa have already been identified for individual regions^8^. Different measures have been enacted to protect VMEs, including spatial and temporal fishing gear restrictions, move-on rules, and areas closed to fishing, but the efficacy of these protection measures has been questioned^9,10^. If VMEs are to be protected adequately by spatial closures then further research is required to improve knowledge of the distribution of VME indicator taxa, to determine if and where genetic diversity hotspots exist, and to quantify the spatial scales at which populations exhibit high levels of genetic diversity and differentiation.

Whilst genetic differentiation amongst many species in the coastal environment is well documented, population genetic structure of deep-sea taxa is much less well elaborated^11^. A limited number of studies, employing a range of genetic markers, have failed to reveal consistent patterns of genetic structuring across a range of deep-sea VME indicator taxa in the New Zealand region^12–16^. The role of currents as either promoters of, or barriers to, gene flow is likely to be region-specific and requires more consideration at a variety of spatial and temporal scales. For example, in waters south of New Zealand, the Antarctic Circumpolar Current is believed to act as an oceanographic barrier to gene flow of deep-sea octocorals, separating populations to the north (around New Zealand and Tasmania) and south (Southern Ocean) at approximately 55°S on the Macquarie Ridge^17^. In addition, deep water (>1000 m) currents flow from north and south off New Zealand’s east coast and meet along the Chatham Rise^18^: such flow may act as a barrier to larval dispersal in some regions and for some taxa^19,20^ but may also promote mixing and genetic diversity in others^16^.

Understanding the spatially dependent patterns of genetic structure (little or no genetic structure is presumed to represent high levels of historical and/or contemporary gene flow, whereas pronounced genetic structure is presumed to represent low or zero levels of gene flow) is important for management planning^2,21^, and the design of a network of MPAs in the New Zealand region^13,15,16^. Whilst deep-sea genetic studies in the New Zealand region span a range of VME indicator taxa, the spatial coverage is patchy and therefore difficult to interpret to inform general protection measures. We examined mitochondrial DNA sequence variation to assess population genetic diversity and differentiation of three deep-sea demosponge species, as well as mitochondrial DNA sequence variation and microsatellite DNA variation for a fourth species, all from the New Zealand region. Sponges are VME indicator taxa in the South Pacific Ocean^8^, and the study species were selected to provide genetic data for a broader project that aims to inform management of VMEs in the South Pacific region. Our study highlights the difficulty that may be faced by researchers of deep-sea taxa in trying to balance the extraction of maximum information from a minimum number of specimens for which genetic markers are not highly informative and/or are difficult to employ. Interpretation provided here will be added to newly published data from VME corals^16,22^ and from other taxa (work in progress) to develop a multi-taxon approach to understanding spatially explicit population genetic variation within and beyond the New Zealand Exclusive Economic Zone (EEZ).

## Results

*COI* sequence data were obtained for all four species, *12S* data for *Penares* sp., and *N*. *persicum*, and *Cytb* data for only *Poecillastra laminaris* (Table S3). Overall, there were no clear and consistent spatially-dependent patterns of *COI* and *Cytb* haplotypic or nucleotide diversity for *Poecillastra laminaris* (Table S4a), for COI and *12S* for *Penares* sp. (Table S4b), for *COI* and *12S* for *N*. *persicum*, or for *COI* for *P*. *menoui* (Table S5). Haplotype accumulation curves failed to reach an asymptote for all markers for *Penares* sp., *N*. *persicum* and *Pleroma menoui* (Fig. S6), indicating that a better understanding of genetic diversity at different spatial scales requires more samples. The haplotype accumulation curves for *COI* and *Cytb* for *Poecillastra laminaris* approached an asymptote despite some haplotypes still being unaccounted for (Fig. S6). These results for *P*. *laminaris* suggest that the sample sizes were large enough to recover large spatial scale genetic diversity but probably too small to recover small spatial scale (e.g., geomorphic feature populations) population structure.

### Geographic distribution of haplotypes

**Figure 1:**
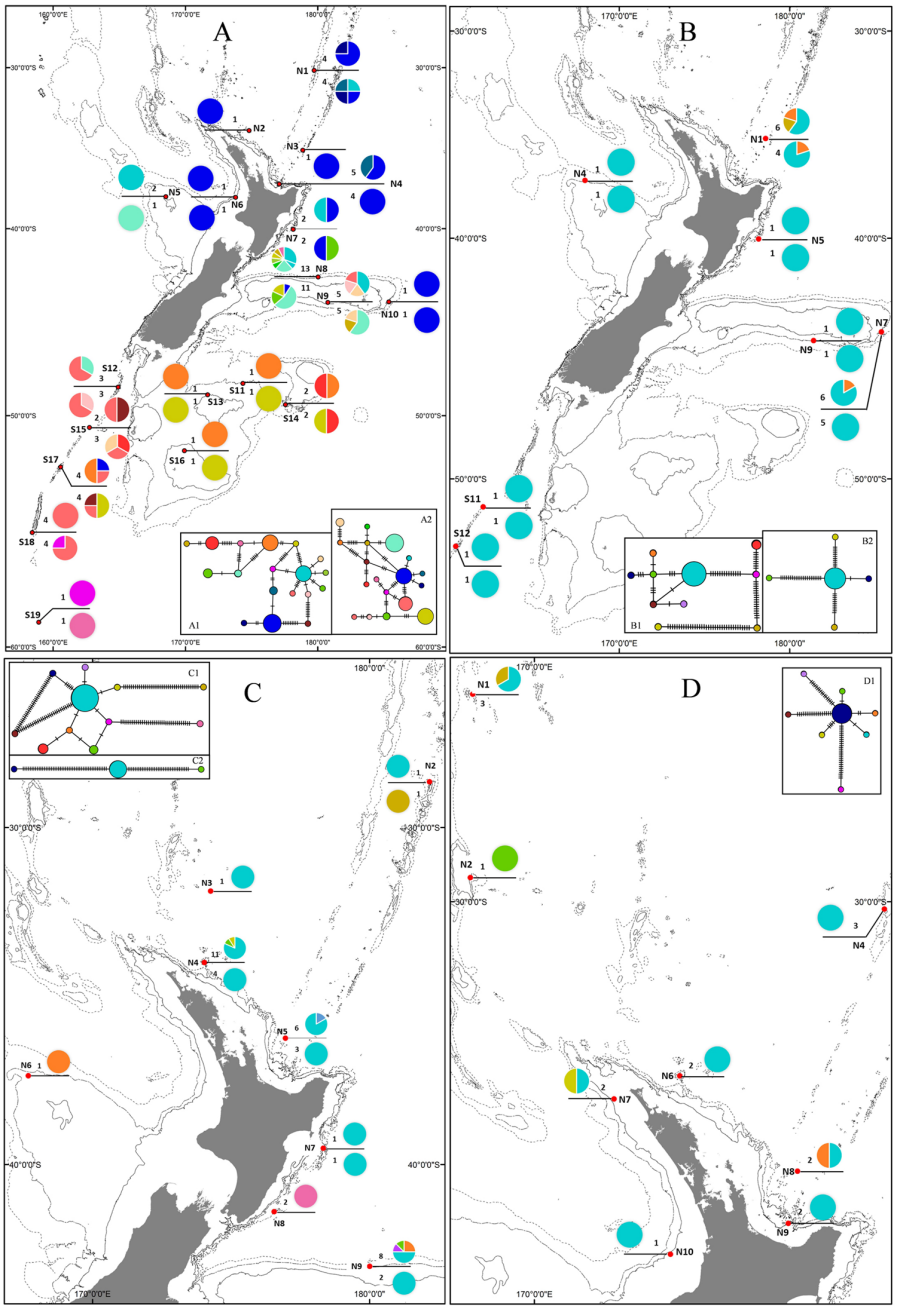
Haplotype maps for *Poecillastra laminaris* - *COI* (above line) and *Cytb* (below line). (**A**) *Penares* sp. *COI* (above line) and *12S* (below line). (**B**) *Neoaulaxinia persicum COI* (above line) and *12S* (below line). (**C**) *Pleroma menoui COI*. (**D**) Insets A1, B1, C1 and D1 are the *COI* haplotype networks for *Poecillastra laminaris*, *Penares* sp., *Neoaulaxinia persicum* and *Pleroma menoui*, respectively. Inset A2 is the *Cytb* haplotype network for *Poecillastra laminaris*, and insets B2 and C2 are the *12S* haplotype networks for *Penares* sp. and *Neoaulaxinia persicum*, respectively.

Eleven *COI* haplotypes were observed for *Poecillastra laminaris* in the northern province, four haplotypes were only observed in the southern province. For *Cytb*, nine haplotypes were distributed only in the northern province and seven haplotypes were only found in the southern province. *Poecillastra laminaris* exhibited clear evidence of spatial genetic differentiation at both markers (Fig. 1A). However, for *Penares* sp., there was low haplotypic diversity for both markers, which was distributed across both provinces. There was no support for the hypothesis of geographic genetic structure by province for haplotypic diversity distributions of *COI* or *12S* for this species (Fig. 1B). There was limited evidence of *12S* haplotypic diversity within the northern province, and the haplotypic distributions of *COI* and *12S* did not reveal patterns of spatial genetic differentiation in *N*. *persicum* (Fig. 1C). Only five *COI* haplotypes of *Pleroma menoui* were found in the northern province, and there was limited evidence to suggest the presence of spatial genetic structure (Fig. 1D).

### Population genetic diversity

For *Poecillastra laminaris*, both *Cytb* and *COI* nucleotide diversity within the southern province was greater than that within the northern province. The central region had the highest nucleotide diversity for *COI* amongst the three regions, whereas nucleotide diversity for *Cytb* in the south was greater than that of the other two regions. Amongst the geomorphic features the highest nucleotide diversity of *COI* and *Cytb* occurred in the Bounty Trough population, although only two sequences were available for two markers (Fig. 1 and Table S4).

For the microsatellite variation of *Poecillastra laminaris*, mean diversity index values (N, Na, Ne, Ho, He) tended to be greater in the northern than in the southern province, whereas mean index values tended to be greater in the south region than in the central or north regions (Table S6). Amongst the four populations for which analyses were possible (Campbell Plateau, Chatham Rise, Kermadec Ridge and Macquarie Ridge), the population associated with the Kermadec Ridge had the highest number of different alleles, of effective alleles, and observed heterozygosity, but the greatest expected heterozygosity was detected in the Macquarie Ridge population.

For *Penares* sp. the *COI* haplotypic and nucleotide diversity values of the southern province were greater than those of the northern province, but it was the opposite in *12S*. This finding is likely to be related to the uneven sample sizes between two provinces. For the three regions, the south had the highest haplotypic and nucleotide diversity values in *COI*, mainly contributed by samples from Macquarie Ridge. The north region had the highest nucleotide diversity in *12S* amongst the regions, which was closely related to high diversity associated with populations from the Kermadec Ridge (Table S4).

For *N*. *persicum* the *COI* haplotypic and nucleotide diversity values were greater in the south than north region, most likely as a result of the uneven sample sizes between the regions. Populations on the Three Kings Ridge had the highest nucleotide and Chatham Rise populations had the highest haplotype diversity values. For *12S* the haplotype number, the number of polymorphic sites, haplotype diversity and nucleotide diversity values could only be calculated for the south region. The Kermadec Ridge population had the highest nucleotide and haplotypic diversity (Table S5).

For *Pleroma menoui*, *COI* haplotypic diversity in the north was greater than in the south region, and amongst the populations it was greatest from the Norfolk Ridge. The nucleotide diversity for *Pleroma menoui* followed the same pattern (Table S5).

### Population genetic structure

For *Poecillastra laminaris* populations, *Φ*_*ST*_ values for *COI* and *Cytb* were highly variable (Table S7), but were often based on small sample sizes (Table S1). AMOVA for this species revealed significant differentiation (p < 0.01) between populations of the northern and southern provinces, amongst populations of the three regions and also amongst populations from the different geomorphic features for both *COI* and *Cytb* (Table 1). Except for a weak significant effect for *Cytb*, populations within provinces, regions or at geomorphic features were not statistically significantly differentiated (p > 0.05), whereas significant differentiation existed within all populations (p < 0.01). The Bayesian phylogenetic trees revealed evidence of geographic structure (Fig. S2). Whilst not all sequences were grouped according to sampling location, most were, providing support for northern and southern differentiation. Based on microsatellite variation, the same scenario as *COI* and *Cytb* was also detected in AMOVA at provincial, regional and geomorphic feature scales (Table 1). Examination of *F*_ST_ values derived from microsatellite variation, revealed that the *F*_ST_ value between the northern and southern provinces was 0.034 (p < 0.05), and the *F*_ST_ values between the north and central, central and south, and north and south regions were 0.033 (p < 0.05), 0.024 (p < 0.05) and 0.052 (p < 0.05), respectively. Amongst the geomorphic features, the Kermadec Ridge population was significantly differentiated (p < 0.05) from the Campbell Plateau and Chatham Rise populations (Table S8).

**Table 1:** Hierarchical AMOVA results for *Poecillastra laminaris* and *Penares* sp. when tested for genetic variation at mitochondrial genes.

Source of variation	*Poecillastra laminaris*									*Penares* sp.					
	*COI*			*Cytb*			Microsatellites			*COI*			*Cytb*		
	df	ss	%V	df	ss	%V	df	ss	%V	df	ss	%V	df	ss	%V
Between provinces	1	40.201	32.34**	1	22.481	21.90**	1	8.184	1.42**	1	0.768	10.53 ns	1	0.012	−22.35 ns
Within provinces between populations	16	60.618	9.53	16	54.323	11.87	51	252.075	11.34**	3	1.600	−0.01	3	0.167	−18.82
Within populations	35	92.068	58.13**	33	76.000	66.23**	53	207.902	87.24**	11	6.257	91.47	9	0.750	141.18
Total	52	192.887		50	152.804		105	468.161		15	8.625		13	0.929	
Amongst regions	2	59.329	36.17**	2	35.060	25.42**	2	17.463	2.45**	2	1.625	18.14 ns	2	0.095	32.24 ns
Within regions between populations	15	41.490	1.32	15	41.744	5.80*	50	243.500	10.74**	4	1.767	16.08	4	0.083	−89.32
Within populations	35	92.068	62.51**	33	76.000	68.7**	53	206.902	86.80**	9	5.233	97.94	7	0.750	157.08
Total	52	192.887		50	152.804		105	467.864		15	8.625		13	0.929	
Amongst geomorphic features	4	68.370	38.58**	4	50.037	34.53**	3	27.553	4.52**	4	2.368	8.06 ns	4	0.179	60.04 ns
Within geomorphic features between populations	11	26.702	−2.15	12	24.596	−3.03	42	199.723	9.94**	2	1.024	−8.89	2	0.000	−119.88
Within populations	34	90.568	63.58**	32	74.000	68.50**	46	177.510	85.54**	9	5.233	100.83	7	0.750	159.85
Total	49	185.640		48	148.633		91	404.786		15	8.625		13	0.929	

df = degree of freedom, ss = sum of squared observations, %V = percentage of total variance. Significant values of p < 0.05 are marked as *, and p < 0.01 are marked as **, and p > 0.05 are marked as ns.

Analyses of *Φ*_*ST*_ values revealed no statistically significant genetic differentiation for *Penares* sp., *N*. *persicum* and *Pleroma menoui* (Tables S9–11). AMOVA revealed no significant (p > 0.05) hierarchical population genetic structure for *Penares* sp. or for *Pleroma menoui* (Tables 1 and 2). AMOVA could not be conducted for *12S* of *N*. *persicum* due to insufficient sequence variation. Bayesian phylogenetic trees and haplotype distributions did not support evidence of significant geographic structure between or within provinces (Figs 1 and S3–5).

**Table 2:** Hierarchical AMOVA results for *Neoaulaxinia persicum* and *Pleroma menoui* when tested for genetic variation at the *COI* mitochondrial gene.

Source of variation	*N. persicum COI*			*P. menoui COI*		
	df	ss	%V	df	ss	%V
Between regions	1	0.133	−25.68	1	0.484	−5.51
Within regions between populations	6	3.564	17.05	6	3.849	25.99
Within populations	22	8.436	108.63	8	3.167	79.53
Total	29	12.133		15	7.500	
Amongst geomorphic features	3	1.44	−18.41	3	1.726	−6.04
Within geomorphic features between populations	4	2.258	27.47	4	2.607	27.45
Within populations	22	8.436	90.94	8	3.167	78.59
Total	29	12.133		15	7.500	

Significant values of p < 0.05 are marked as *, and p < 0.01 are marked as **.

### Isolation-by-distance and depth

Tests of isolation-by-distance and isolation-by-depth were performed for data sets where there was no evidence of geographic genetic differentiation, that is, for all species excluding *Poecillastra laminaris*. The 10 tests across the three species revealed no significant instances of isolation-by-depth or isolation-by-distance, except an isolation-by-depth signature for *COI* in *N*. *persicum* (Table 3).

**Table 3:** Mantel tests of isolation-by-depth and isolation-by-distance amongst different markers and species.

Species	Depth		Distance	
	*COI*	*12S*	*COI*	*12S*
*Penares* sp.	0.169 (0.060)	0.114 (0.240)	0.103 (0.280)	−0.092 (0.610)
*Neoaulaxinia persicum*	**−0**.**049** (0.039)	−0.202 (0.170)	0.156 (0.060)	0.554 (0.070)
*Pleroma menoui*	0.001 (0.460)		−0.032 (0.520)	

Values shown as correlation coefficient (p value). Numbers with bold mean significant isolation. *Poecillastra laminaris* was not tested because of pronounced genetic structure at different spatial scales.

### Location of genetic discontinuities

**Figure 2:**
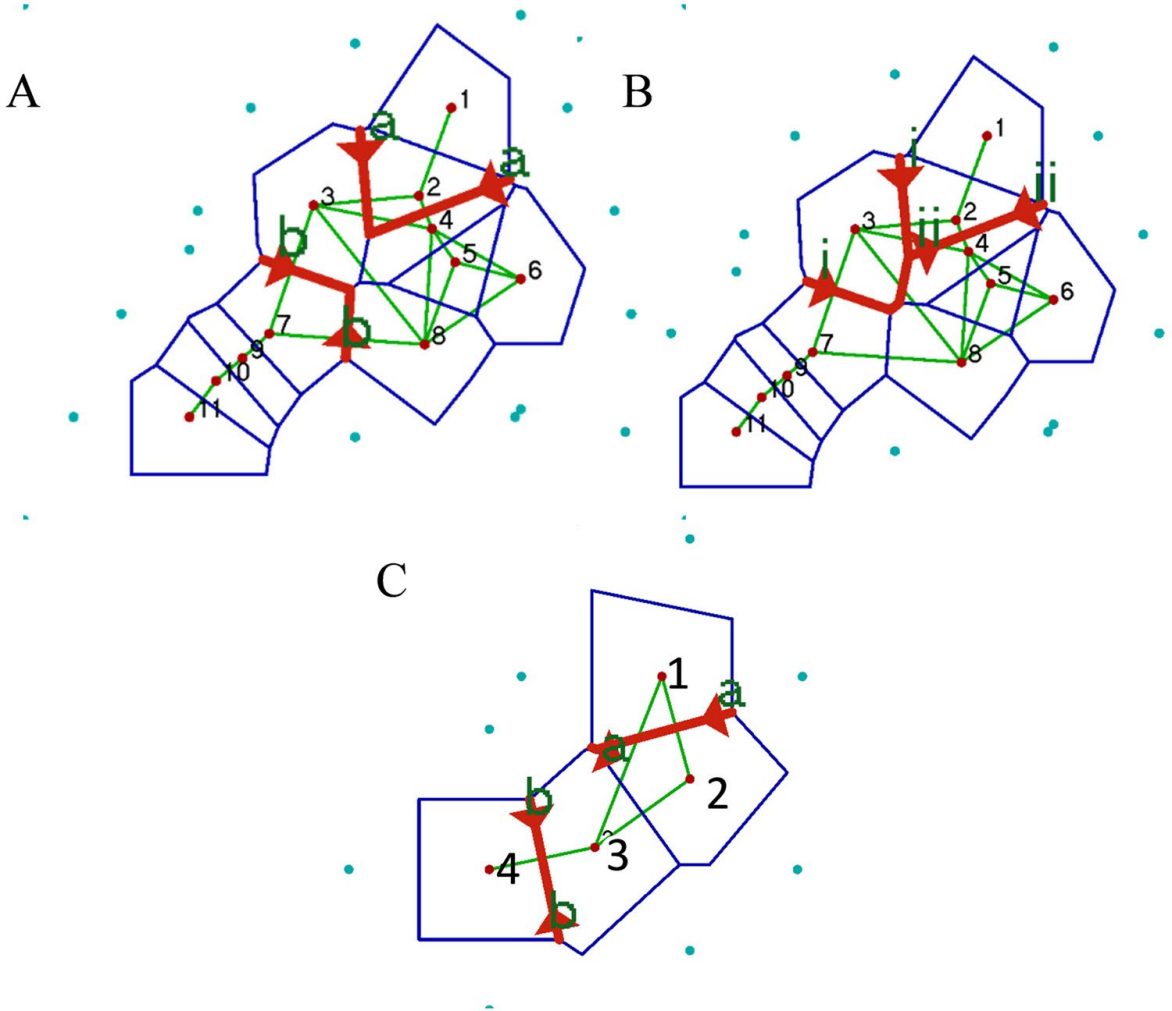
Predicted locations of genetic discontinuities (red lines) for *Poecillastra laminaris* populations based on *COI* variation (**A**) *Cytb* variation (**B**) and microsatellite variation (**C**) from Barriers software. Geomorphic populations were pooled in (**A**,**B**) 1–2 are Kermadec Ridge; 3 is Challenger Plateau; 4 is Hikurangi Margin, 5–6 are Chatham Rise; 8 is Campbell Plateau; 7–11 are Macquarie Ridge. In panel C, geomorphic population labelled 1 is Kermadec Ridge; 2 is Chatham Rise; 3 is Campbell Plateau, 4 is Macquarie Ridge.

Locations of genetic discontinuities between/amongst populations of *Poecillastra laminaris* based on the *COI* pairwise population *Φ*_*ST*_ values and sampling site geographical coordinates were predicted to occur around the north of the North Island and the south of the South Island of New Zealand (Fig. 2A). Based on the *Cytb Φ*_*ST*_ values the analysis indicated that discontinuities may also exist to the west and north of the North Island (Fig. 2A,B). Genetic discontinuities around the north of the North Island and the south of the South Island were detected based on the microsatellite *F*_ST_ values (Fig. 2C).

### Migration and connectivity

Estimates of *θ* and larval migration rates were only calculated for *Poecillastra laminaris* because this data set was the largest and had the best spatial coverage Average *θ* values estimated for the combined *COI* and *Cytb* data set ranged from 0.0001 (Kermadec Ridge) to 0.0079 (Hikurangi Margin) (Table 4). The estimates of per generation migrants were variable amongst samples. For populations in the northern province (those on the Kermadec Ridge, Challenger Plateau and Hikurangi Margin) there was no migrant contribution to populations in the southern province (populations on the Campbell Plateau and Macquarie Ridge), but the Macquarie Ridge population contributed migrants to the Kermadec Ridge and Challenger Plateau populations. The Kermadec Ridge was the only source of migrants to the Hikurangi Margin population, and the Chatham Rise population (in the central region) contributed to all populations except that on the Hikurangi Margin, and received migrants from all populations except those in the south region (Table 4).

**Table 4:** Theta (*θ*) and number of effective migrants per generation amongst pooled populations from 6 regional locations based on combined *COI* and *Cytb* sequences for *Poecillastra laminaris*.

	To Kermadec Ridge	To Challenger Plateau	To Hikurangi Margin	To Chatham Rise	To Campbell Plateau	To Macquarie Ridge
From Kermadec Ridge (North region)	0.0001	156.80	2360.00	470.37	—	—
From Challenger Plateau (North region)	1010.00	0.0027	—	417.08	—	—
From Hikurangi Margin (North region)	22.39	—	0.0079	89.57	—	—
From Chatham Rise (Central region)	464.27	2230.00	—	0.0067	650.04	557.16
From Campbell Plateau (South region)	—	—	—	—	0.0063	582.17
From Macquarie Ridge (South region)	314.76	366.92	—	—	1420.00	0.0042

For migration occurring between population pairs, the top row is the receiving population (To population) and the left column is the broadcasting population (From population). *θ* values are given on the diagonal.

Only migrant values >1 are shown (values <1 are shown as - in the table).

## Discussion

It is still unusual for managers to explicitly consider patterns of genetic diversity in the conservation of marine resources^2,23^, despite the fact that the value of such information for spatial planning and conservation prioritisation has long been appreciated^24^. This situation may be because few guidelines or frameworks exist to help managers with the incorporation of genetic data into their planning, although recent work has started to address this gap^2,23^. Whilst predictive habitat suitability modelling for VME indicator taxa in the New Zealand region^25,26^ has been developed to inform spatial management processes, to date this has not included the input of genetic data. In the present paper, a multi-species approach has been employed to improve our understanding of spatially explicit deep-sea genetic diversity and genetic structure, to identify the location of genetic discontinuities (putative barriers to gene flow) within the New Zealand region, and to identify patterns of connectivity and dispersal amongst populations of demosponges.

A major constraint of studies of deep-sea genetic connectivity is often the limited availability of material, both numbers of individuals per population and numbers of populations at a range of depths^11,13,15^. Using archived material collected over several decades from many regions of the New Zealand EEZ we started with reasonably large samples sizes and with good spatial coverage, but preliminary testing of this material soon revealed that much of it could not be used for genetic analyses, consistent with earlier work on VME vent mussels^15^. DNA degradation of these historic samples did not only reduce sample sizes, it also hindered the development of DNA markers such as microsatellites for some species, and SNPs for all species. Nonetheless, because sponges are important VME taxa, and because the collection of new material is unlikely to happen in the near future, we proceeded to analyse the workable material from the four sponge species, whilst recognising limitations to the data set. In this study, small sample sizes for all sponge populations inhabiting different sites may fail to reveal the full extent of genetic diversity within such populations. For example, (1) the total sample size was ≥30 individuals for only *Poecillastra laminaris* and *N*. *persicum*, (2) haplotype accumulation analyses showed that all four species did not have enough specimens to recover all expected haplotypic diversity, and (3) in several cases individual population sample sizes were small (arbitrarily set minimum of 4 individuals for all analyses). Nonetheless, a fuller understanding of the population genetics of many deep-sea taxa must be pursued with less than perfect sample sets because of limited sampling opportunities, and the urgent management need to protect biodiversity, in particular VMEs. Hence, studies such as the present one, and that of Boschen *et al*.^15^, attempt to minimise the biases of small sample sizes by using conservative analytical approaches and qualitative interpretations of genetic structure and patterns of connectivity.

Compared with shallow water sponges, studies of the population genetic variation of deep-sea sponges are uncommon, but typically report that the genetic diversity of the mitochondrial genome in demosponges is low and that rates of evolution are slow^27–29^. Consistent with these findings, much of the data generated by this study revealed low levels of mitogenome variation. Nonetheless, we observed sufficient genetic variation within and amongst the four species to reveal patterns of genetic structure of sufficient magnitude to be useful to inform management options. Below, we place the patterns or absence of genetic structure for sponges in a broader context, and then we relate this information to conservation management and the establishment of MPAs.

Several patterns of genetic structure amongst the four demosponges were revealed by mtDNA markers within and between the two lower bathyal biogeographic provinces in the New Zealand region. *Penares* sp. exhibited an apparent absence of regional genetic differentiation between the provinces, indicative of high levels of gene flow and consistent with results for highly mobile deep-sea species, including fish^30,31^ and giant squid^32^. Sponges are sessile and one possible explanation for panmixia is that *Penares* sp. larvae may have a long dispersal duration that allows the species to overcome physical barriers between the two provinces (see section below). In addition, the present-day distribution of lithistids probably reflects palaeo-environmental change around the time of the Eocene, whereas astrophorina such as *Poecillastra laminaris* and other non-lithistids are not so constrained^19,20^. Elsewhere, it has been reported that genetic variability in mitochondrial markers is species-dependent in shallow water sponges, with some, but not all, species exhibiting variability over relatively short geographic distances^33–35^. Whether this is true of deep-sea sponges remains unknown, given the relatively poor state of knowledge about the genetics of this group.

Between-province genetic structure was observed for *Poecillastra laminaris*, which is consistent with low or no gene flow between the northern and southern provinces (indicated by both *COI* and *Cytb*). Similar large-scale north-south genetic structure has been reported for corals^13^, fish^36^, and a sea star^37^. Within the two biogeographic provinces, gene flow was evident for all four sponges, even at scales of 1000 s of kilometres. Similar levels of gene flow, with little or no evidence of within-province structure in the same region, have been observed amongst populations of fish^38^, ophiuroids^39^ and crustaceans^9^. Other studies in the region have revealed evidence of genetic structure at smaller spatial scales within provinces, for example for deep-sea amphipods^40^ and polychaetes^9^.

Species-specific differences in reproductive strategies may contribute to differences in patterns of gene flow and regional genetic structure^9^. Deep-sea demosponges have a wide-range of reproductive strategies^41^, but no studies have determined empirically the reproductive strategies or modes of larval dispersal for the four species examined in the present study. Studies of other demosponges have reported asexual and sexual reproduction, as well as dispersal by larvae and by floating propagules, and these different strategies corresponded directly with the genetic connectivity patterns amongst different species^41^. At least eight different larval types are recognised within the Phylum Porifera but the form of those in the suborder Astrophorina, order Tetractinellida, within which our four sponge species are classified, is unknown^42^. Sponge larvae are generally considered to be lecithotrophic, ciliated and thus mobile to some extent, but with a relatively short (indirect development) or non-existent (direct development) planktonic life^42^. This limited dispersal potential may result in clustered distributions of sponges^43^. Detailed information about the reproductive biology of the four species studied here would be highly beneficial for assessing the importance of reproductive and dispersal strategies to population structure and genetic connectivity.

The northern and southern province-level of genetic differentiation for *Poecillastra laminaris*, and the within-province pattern of genetic homogeneity reported for *Poecillastra laminaris*, *Pleroma menoui* and *N*. *persicum*, may be attributable to oceanic current systems. Currents may promote the long-distance transport of larvae, but may also act as barriers to gene flow or cause retention of larvae^44,45^. In the New Zealand region, the major currents with potential to influence population connectivity are associated with the Tasman Front, the Subtropical Front and the Subantarctic Front (Fig. S7). The different currents and frontal systems give rise to different environments in the north and south of the region, and may impede larval dispersal and reduce, or block, gene flow across the Chatham Rise. The haplotypic distribution of *Poecillastra laminaris* supports the contention that gene flow into and through the region may be affected by ocean currents, and indicates that two main population connection routes exist. Firstly, a northern route from the northwest of the North Island with the southerly flow of the East Auckland Current down the east coast of the North Island with the East Cape Current, and then eastwards immediately north of the Chatham Rise and the Subtropical Front; and secondly, a southern route from south of the South Island northwards with the Southland Current, then eastwards immediately south of Chatham Rise and the Subtropical Front. The unique *Poecillastra laminaris COI* and *Cytb* haplotypes found at the most southerly location in the region (Site S19, Fig. 1A) may indicate that populations in this area are isolated by the Antarctic Circumpolar Current and the Subantarctic Front. Further evidence is needed to support this hypothesis for *Poecillastra laminaris*, but a recent study indicates that this current is capable of separating and structuring octocoral populations, and it acts as a ‘soft’ barrier^17^. This interpretation is also supported by studies of the present-day vs palaeoenvironmental distribution of some Recent and fossil lithistid sponges^19,20,46^.

Whilst depth may be an important factor contributing to patterns of genetic connectivity for some benthic fauna^14,39^, we found no evidence for isolation-by-depth. However, small samples sizes have reduced the power to detect such a relationship. In addition, the deepest specimen of any of the four species was collected from ~1500 m, whilst depth-related distinctions in population structure reported in most previous studies were deeper: 3000 m for the amphipod *Eurythenes gryllus*^47^, 2500 m for the bivalve *Deminucula atacellana*^48^, and 1700 m for ophiuroid species^39^. However, in the New Zealand region, Miller *et al*.^14^ reported that populations of the stony cup coral *Desmophyllum dianthus* from different depth strata were strongly differentiated, indicating limited vertical larval dispersal. These authors suggested that this genetic differentiation with depth was consistent with the stratification of different water masses in the region. Further sampling across a greater depth range within the New Zealand region is required to test the existence of a relationship between depth and genetic differentiation for the four sponges examined in the present study, but at the moment there is no evidence to support isolation-by-depth.

**Figure 3:**
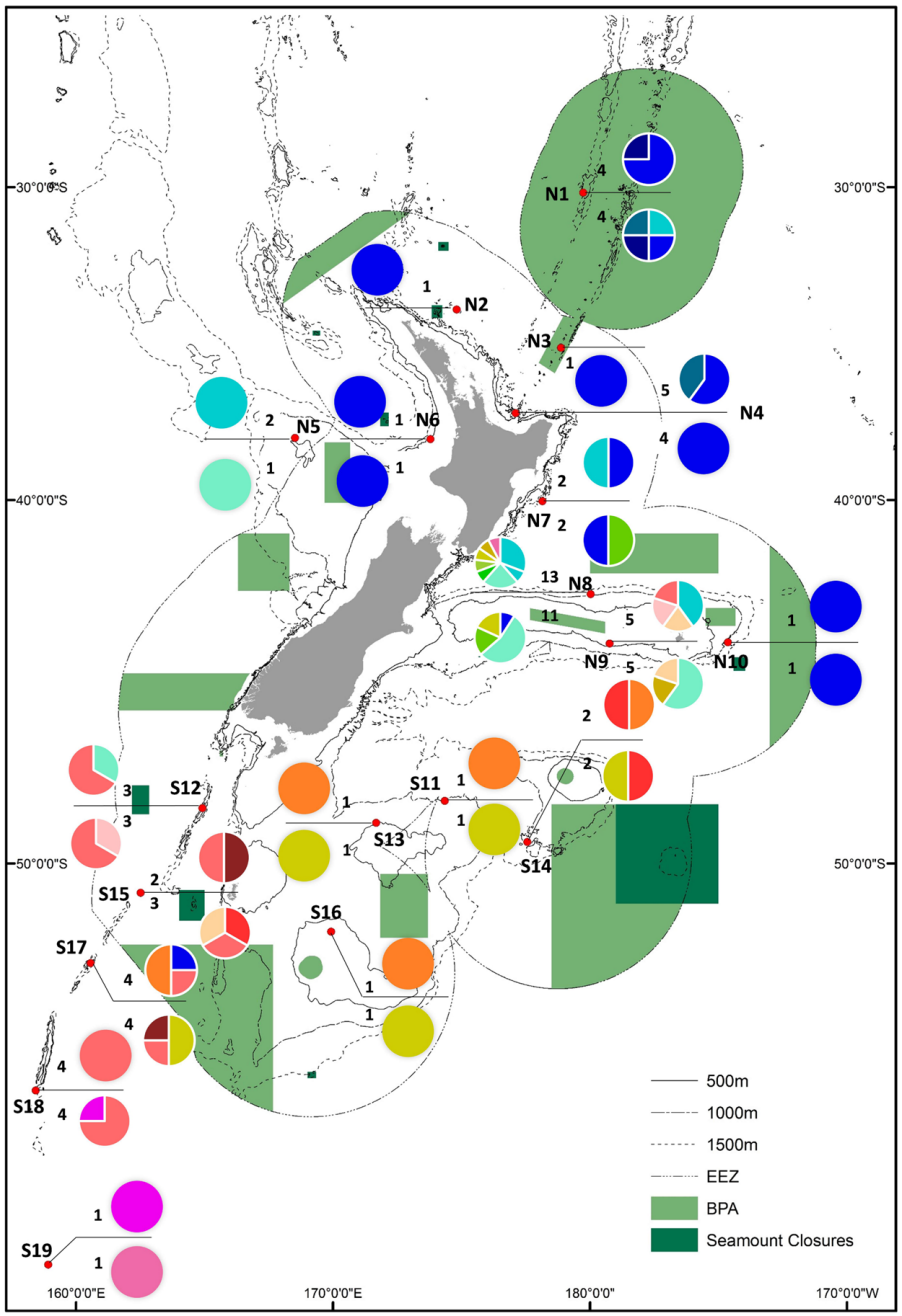
Haplotype map for *Poecillastra laminaris COI* (above line) and *Cytb* (below line) overlain with marine protected areas (BPAs and Seamount Closure Areas) within the New Zealand EEZ. Pie graph indicates haplotypic composition of each location and numbers indicate total number of sequences from each location.

Increasingly, genetic data are now being considered in the decision-making process around marine conservation^2,15,23^. A recent review^2^ highlights how different genetic metrics such as diversity, uniqueness, distinctness of sites and of regions may be applied to management options (the conservation response) depending on if the objective is to conserve biodiversity or to maintain function. In the context of the present work, the primary focus is on the conservation of biodiversity, with a secondary focus on the maintenance of VME function. Protection of deep-sea habitats in New Zealand is afforded to date by 17 seamount area closures (protecting 19 seamounts) and 17 Benthic Protection Areas (BPAs) which were established throughout the New Zealand EEZ in 2001 and 2007, respectively^49,50^ (Fig. 3). These areas are designed to protect benthic fauna by prohibiting trawling on the seafloor (and any form of fishing within 50 m of the seafloor). Amongst the criteria for the selection of these protected areas was that they include fauna vulnerable to disturbance from bottom trawling (i.e., VMEs) and are representative of the benthic fauna and habitats found throughout the EEZ. However, the genetic connectivity of the fauna within and between these areas was not considered in their selection. Since their establishment, only two studies have examined the efficacy of the BPA design, and both have suggested that the location of the BPAs need to be improved to provide more effective protection of biodiversity^9,51^.

Understanding connectivity between and amongst areas is fundamental to designing an effective MPA network, or for modifying the existing distribution of protected areas to create a network^23,24^. Protection of genetically distinct populations should also be considered as an integral component of protected area design in order to maintain genetic diversity^9^. The results of the present study indicate that populations of *Penares* sp., *Pleroma menoui* and *N*. *persicum* are likely to be well connected amongst the presently protected areas. Data for *Poecillastra laminaris*, the most widely sampled species, indicates that populations associated with the Kermadec Ridge, Challenger Plateau, Chatham Rise and the Macquarie Ridge may be the main genetic sources for populations around New Zealand. The Kermadec BPA provides protection for the northern source of genetic population connectivity. This large BPA has recently been proposed for further protection as an ocean sanctuary (http://www.mfe.govt.nz/marine/kermadec-ocean-sanctuary/question-answers-kermadec-ocean-sanctuary), although the final decision on this proposal is still pending. The Challenger Plateau has two BPAs that cover deep water to the south, and a shallower area on the top of the plateau which will provide some protection for genetic sources on the Challenger Plateau. However, there are no BPAs covering any part of the Macquarie Ridge, although two seamount closures to the west and east of the ridge, and a BPA to the south, may provide some protection for the southern source of genetic connectivity. There are protected areas in relatively close proximity to the remainder of the study sites that represent the range of genetic variation observed for *Poecillastra laminaris* across the region. However, whether these areas protect this diversity will depend on whether they provide suitable habitat for VME indicator taxa such as sponges.

Our findings indicate that additional protected areas on the Chatham Rise should be considered for sponges. The two BPAs on the Chatham Rise cover a very limited depth range *along* the Rise (west-east), and this may not be sufficient to protect the genetic variation observed *across* the Rise (north-south). Whilst there are no population genetic data for *Poecillastra laminaris* from within these two BPAs, the haplotypic distributions showed the Chatham Rise population had the highest genetic diversity of all populations sampled in the study. This suggests that further protected areas, or an enlargement of the current BPAs, need to be considered to afford greater protection to the benthic populations associated with the Chatham Rise. This recommendation was also made by Bors *et al*.^9^ for other benthic taxa studied on the Chatham Rise.

Many countries, New Zealand included, have international obligations to protect deep-sea VMEs, but the decision-making process about the placement, size and spacing of MPAs is often hindered by a lack of understanding about genetic diversity and connectivity of VME taxa. Using historically collected samples and a small data set, we demonstrate how archived material may be employed to inform management decisions about the protection of deep-sea VMEs. The results of the present study, and those of previous genetic connectivity studies around New Zealand^9,12,13,15–17,23,52^, are now beginning to provide information that can be used to improve the design of protected areas (such as BPAs and seamount closure areas) in the New Zealand EEZ and beyond. The results from these studies demonstrate the need for a flexible spatial management system that can be periodically adjusted to accommodate increased understanding about the connectivity of a range of benthic taxa in the region as further studies are conducted.

## Material and Methods

### Samples

Four astrophorinid demosponge species (suborder Astrophorina Sollas) were analysed (Fig. S1 and Table S1): *Neoaulaxinia persicum* Kelly, 2007 (family Phymatellidae Schrammen), an as yet, undescribed species of *Penares* sp. (family Geodiidae Gray), *Pleroma menoui* Lévi & Lévi 1983 (family Pleromidae Sollas), and *Poecillastra laminaris* Sollas 1886 (family Vulcanellidae). Specimens were identified using a combination of standard morphological taxonomic techniques^53^ and molecular information. Samples were sourced from the NIWA Invertebrate Collection (NIWA, Wellington). The majority of specimens were from seamount and slope habitats from 122–1507 m water depth. Most were preserved in ethanol, the rest dry-preserved.

### Spatial structure of sample allocation

Samples were assigned to different populations based on the highest level of spatial differentiation, with the result that individuals of *N*. *persicum* (Σn = 30), *Penares* sp. (Σn = 16), *Pleroma menoui* (Σn = 16) and *Poecillastra laminaris* (Σn = 55) were assigned to 6, 5, 4 and 8 different geomorphic feature populations, respectively.

**Figure 4:**
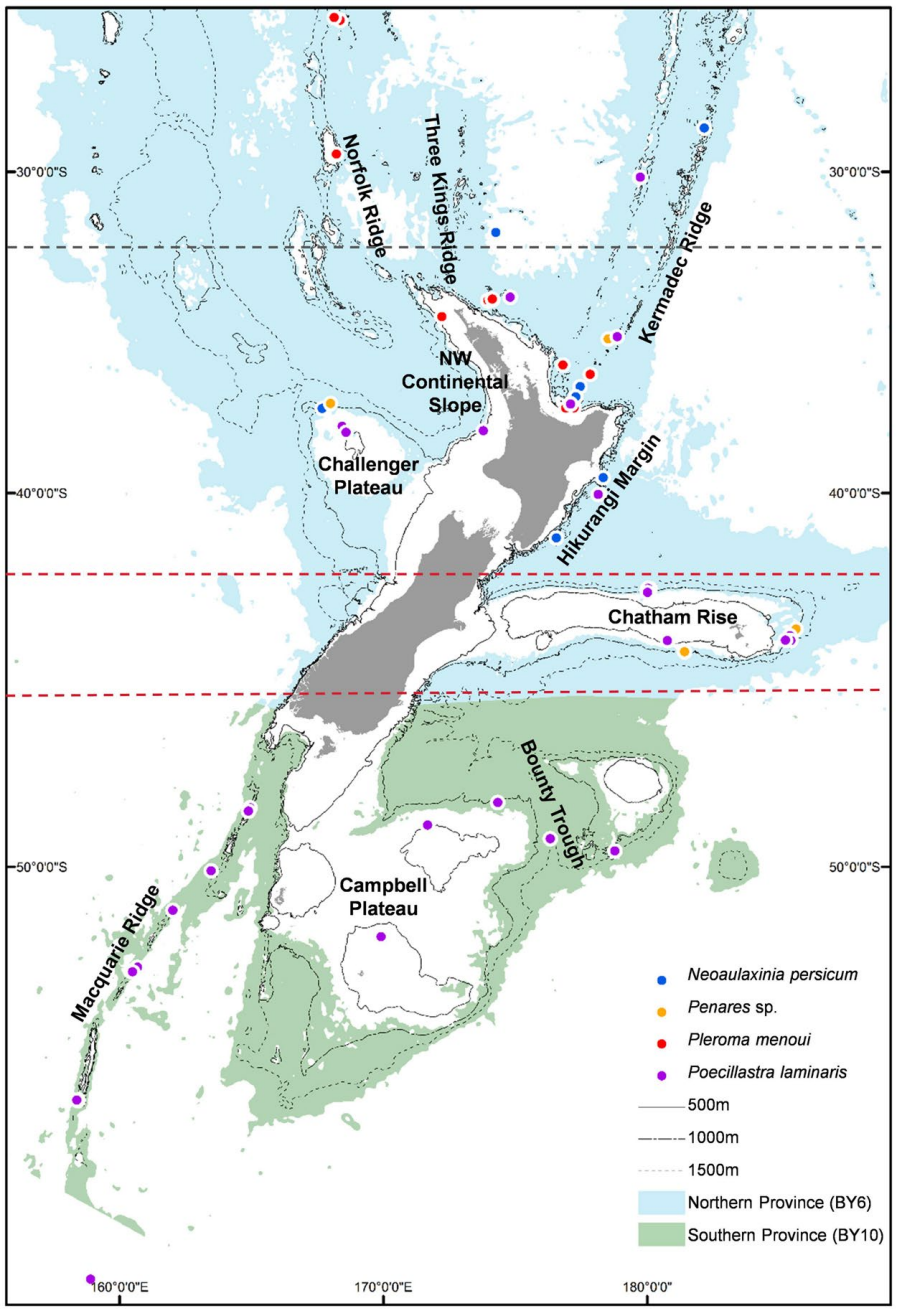
Map showing the locations of samples for the study species. Hierarchical testing of regional genetic differentiation is employed: (1) blue (northern) and green (southern) backgrounds represent the two biogeographic lower bathyal provinces (BY6 and BY10) of Watling *et al*.^54^ in the New Zealand region; (2) red dashed lines at 42°S and 45°S indicate boundaries between north (<42°S), central (42°S to 45°S) and south (>45°S) regions for *Poecillastra laminaris* and *Penares* sp. and black dashed line at 32°S shows boundary between north and south regions for *Neoaulaxinia persicum* and *Pleroma menoui*; (3) major geomorphic features as named on the map.

Patchy deep-sea sampling and the different distributions of the four species meant that samples were not available for all species from all locations^19,20^ (Fig. 4 and Table S1). To achieve a balance between the validation of results and extracting maximum information content from the specimens, the minimum sample size was set at four for population analysis. For hypothesis testing we employed a biogeographic province, region and geomorphic features hierarchical framework: (i) water mass characteristics of the two New Zealand deep-sea biogeographic provinces^54^ affect population distributions, which results in a pattern of province-scale genetic structure; (ii) northern and southern currents that meet and mix to the east along the Chatham Rise and which give rise to the Subtropical Front may influence larval dispersal, which results in a north–central–south regional-scale pattern of genetic structure and increased genetic diversity on the Chatham Rise; and (iii) current flows, eddies and turbulent mixing associated with topographic features (such as seamounts, plateaux, rises, ridges, slopes, troughs, basins) may restrict larval dispersal amongst such features, thereby contributing to genetic differentiation amongst populations on the features (Fig. 1). For *N*. *persicum* and *Pleroma menoui* (only available from the northern province), a further division at ~32°S (the East Auckland Current flows over the northern province^18^), was applied at a regional level. Tests were also conducted to examine the influence of geographic distance and depth on genetic population structure. Where appropriate, *post hoc* analyses were conducted to estimate effective population size (*N*e), the location of genetic discontinuities independent of the hypothesis testing framework, and to identify patterns of migration.

### Molecular methods

Three mitochondrial DNA regions, the *12S rRNA* (*12S*), *cytochrome c oxidase subunit I* (*COI*) and *cytochrome b* (*Cytb*), were used to estimate genetic diversity and population genetic differentiation. Sponge-specific primers for *12S*, *COI* and *Cytb* were developed from the *Poecillastra laminaris* mitochondrial genome using Primer 3.2 in Geneious, and amplifications were tested for all four species as described by Zeng^55^ (Tables S2 and S3). Microsatellite (nuclear DNA) data were only available for *Poecillastra laminaris*. DNA extraction, PCR amplification conditions and genotyping details are described by Zeng^55^.

### Data analyses

DNA sequences were checked and edited and multiple sequences were aligned using ClustalW Alignment in Geneious (v7, Biomatters Ltd, New Zealand). Representative haplotypes have been deposited in GenBank (Table S3). Bayesian analysis was performed using MrBayes 3.2.6 plugin in Geneious using invgamma rate variation and gamma categories set to 4, and highest similarity sequence in GenBank as an out-group. The first 50% of a 1,000,000 chain length was discarded as burn-in, and 4 heated chains were run with a subsampling frequency of 1000. Intra-specific genetic diversity was evaluated for all populations with two or more individuals by computing the number of haplotypes, the number of polymorphic sites, haplotypic diversity (*h*), and nucleotide diversity (*π*) using DnaSP v5^56^. Minimum spanning haplotype networks were drawn using Popart (v1.7, University of Otago) Specimens were colour-coded according to haplotype and their geographic coordinates of collection were plotted by ArcGIS (ESRI, USA).

Analysis of molecular variation (AMOVA) was tested between/amongst locations (northern versus southern biogeographic provinces; north versus central versus south regions; amongst geomorphic features) and amongst populations using Arlequin^57^. The Mantel test was employed to test for isolation-by-depth and isolation-by-distance by comparing the matrix of Nei’s unbiased genetic distances (generated by MEGA v6^58^ to the matrix of depth (m) and the matrix of shortest actual distances (km) between pairs of sites using GenAlEx v6.5^59^.

Pairwise comparisons of population differentiation were performed in Arlequin and significance values estimated after 1,000 permutations. Between-province or within-province *Φ*_*ST*_ statistics were calculated to test for genetic differentiation amongst populations. If significant differentiation amongst populations was detected, the location of the genetic discontinuity was identified using the software Barrier v2.2^60^.

Migrate-n was used to estimate *θ* (effective population size × mutation rate per site), and migration (M) under the sequence model^61^. Because of small sample sizes for 3 of the 4 species, values were only estimated for *Poecillastra laminaris*. Average *θ* values and migration values were estimated for the combined *COI* and *Cytb* data set. A full migration matrix model was employed as the migration estimating model, and each Markov chain Monte Carlo (MCMC) run consisted of 10 short chains (sampling 50,000 trees) and one long chain (sampling 500,000 trees) with a burn-in period of 10,000 trees.

To assess differential sampling efforts, haplotype accumulation curves were generated by calculating estimates of the mean and variance for the number of accumulated haplotypes through 1,000 random permutations, using the R package SPIDER^62^.

Microsatellite diversity was estimated in GenAlEx, including allelic frequencies, number of alleles, and observed and expected heterozygosities. Arlequin was used to estimate Weir and Cockerham’s (1984) unbiased estimator of Wright’s *F* statistic (*F*_ST_). Population genetic differentiation was tested using Arlequin (AMOVA, 10,000 permutations) for differentiation between provinces, amongst regions and amongst geomorphic features.

The Mantel test (GenAlEx) was employed to test for isolation-by-depth by comparing the matrix of individual-based genetic distance values (generated by GenAlEx) to the matrix of depth (m) values and isolation-by-distance by comparing the matrix of individual-based genetic distance values to the matrix of shortest actual distances (km) between pairs of sites. These analyses were conducted for all species at the largest spatial scale at which significant population genetic differentiation was not observed.

## Supplementary information

Supplementary Information for the use of spatially explicit genetic variation data from four deep-sea sponges to inform the protection of Vulnerable Marine Ecosystems

## Data Availability

Sequence data have been archived in the NCBI database, and details of sequences and sampling information are listed in the Supplementary Materials Section.
