# Supplementary material for: The use of spatially explicit genetic variation data from four deep-sea sponges to inform the protection of Vulnerable Marine Ecosystems
Source: Sci Rep. 2019 Apr 2;9:5482. doi: 10.1038/s41598-019-41877-9 (PMC6445101; doi:10.1038/s41598-019-41877-9)
Supplement: Supplementary file 1 — Supplementary Information for the use of spatially explicit genetic variation data from four deep-sea sponges to inform the protection of Vulnerable Marine Ecosystems [file 41598_2019_41877_MOESM1_ESM.pdf]

Supplementary Information

The use of spatially explicit genetic variation data from four deep-sea sponges to inform the protection of Vulnerable Marine Ecosystems

Cong Zeng[1,2,3*], Malcolm R. Clark[3], Ashley A. Rowden[2,3], Michelle Kelly[4], Jonathan P. A. Gardner[2]

1 - College of Animal Science and Technology, Hunan Agricultural University, Changsha, China
2 –School of Biological Sciences, Victoria University of Wellington, Wellington 6140, New Zealand
3 - Coasts and Oceans National Centre, National Institute for Water and Atmospheric Research, Private Bag 14901, Kilbirnie, Wellington, New Zealand
4 - Coasts and Oceans National Centre, National Institute of Water and Atmospheric Research, Auckland 1010, New Zealand
*Corresponding Author: congzeng@live.cn

Supplementary information

Table S1. Details of collections of and sample sizes for four demosponges

Northern-Southern biogeographic provinces	North-Central-South regions	Geomorphic features	*Neoaulaxinia persicum* (n)	*Penares* sp. (n)	*Pleroma menoui* (n)	*Poecillastra laminaris* (n)
Northern	North	Norfolk Ridge			4	
		Three Kings Ridge	1			
		Kermadec Ridge	7	5	7	11
		NE Continental Slope	11		2	1
		NW Continental Slope			3	1
		Challenger Plateau	1	1		2
		Hikurangi Margin	2	1		2
	Central	Chatham Rise	8	7		19
Southern	South	Campbell Plateau				5
		Macquarie Ridge		2		14

(n) – number of individuals

Table S2. Details of PCR primers and conditions used to amplify three mitochondrial DNA regions

DNA region	Primer name	Primer 5'-3'	Annealing temperature (°C)	Trimmed size (bp)
12S	Rnl F	GACACAGGGATGTCGCAGAA	50	~700
	Rnl R	TGACGGGCGATTTGTACGAA		
COI	19F	TGGTGCTTTTTCGGGGATGA	50	400-470
	570R	ATCGCACCAGCCAATACAGG		
Cytb	Cytb F	CGCTAATGGGGCGTCTATGT	50	402
	Cytb R	AAAGTTTTCTGCGTCCCCCA		

Table S3. Number of partial sequences (n) plus length of sequence (bp) for three mitochondrial genes for each of the four sponges

Genes	Species	n	length	NCBI Accession ID
COI	*Poecillastra laminaris*	54	402	Hap_1-18: KX685272-KX685289
	Penares sp.	16	423	Hap1_5: KX685302-KX685306
	Neoaulaxinia persicum	30	462	Hap1_7: KX685290-KX685296
	Pleroma menoui	16	431	Hap1_5: KX685297-KX685301
CytB	*Poecillastra laminaris*	49	402	Hap_1-18: KX685307-KX685324
12S	*Penares* sp.	14	694	Hap_1-2: KX685325-KX685326
	Neoaulaxinia persicum	11	704	Hap_1-2: KX685327-KX685328

Table S4. Nucleotide diversity statistics for *Poecillastra laminaris* at three different spatial scales

Grouping		*COI*				*Cytb*			
		N	n	Hd	π	N	n	Hd	π
Biogeographic Province	Northern	34	14	0.848	0.0145	31	11	0.800	0.0120
	Southern	19	7	0.749	0.0156	19	8	0.811	0.0152
Region	North	15	4	0.619	0.0037	14	6	0.681	0.0054
	Central	19	12	0.895	0.0185	17	7	0.721	0.0142
	South	19	7	0.749	0.0156	19	8	0.811	0.0152
Geomorphic feature	Kermadec Ridge	9	3	0.556	0.0021	9	4	0.583	0.0017
	Challenger Plateau	2	1	0	0	2	1	0	0
	Hikurangi Margin	2	2	1.000	0.0075	2	2	1.000	0.0100
	Chatham Rise	19	12	0.895	0.0185	17	7	0.721	0.0142
	Bounty Trough	2	2	1.000	0.0473	2	2	1.000	0.0274
	Campbell Plateau	3	1	0	0	3	1	0	0
	Macquarie Ridge	13	5	0.628	0.0121	14	7	0.758	0.0137
Total		53	18	0.893	0.0185	50	18	0.889	0.0152

N is the number of sequences for analysis from the site, n is the number of haplotypes detected in the examined site, Hd is haplotype diversity, and π is nucleotide diversity.

- data are unavailable.

Table S4. Nucleotide diversity statistics for *Penares* sp. at three different spatial scales

Grouping		*COI*				*12s*			
		N	n	Hd	π	N	n	Hd	π
Biogeographic Province	Northern	14	4	0.396	0.0025	12	2	0.167	0.0002
	Southern	2	2	1.000	0.0047	2	1	0	0
Region	North	7	3	0.524	0.0041	6	2	0.333	0.0005
	Central	7	2	0.286	0.0007	6	1	0	0
	South	2	2	1.000	0.0047	2	1	0	0
Geomorphic feature	Kermadec Ridge	5	3	0.700	0.0052	4		0.500	0.0004
	Challenger Plateau	1	1	-	-	1	1	-	-
	Hikurangi Margin	1	1	-	-	1	1	-	-
	Chatham Rise	7		0.286	0.0007	6	1	0	0
	Bounty Trough	-		-	-	-		-	-
	Campbell Plateau	-		-	-	-		-	-
	Macquarie Ridge	2	2	1.000	0.0047	2	1	0	0
Total		30	5	0.411	0.0025	26	2	0.148	0.0002

N is the number of sequences for analysis from the site, n is the number of haplotypes detected in the examined site, Hd is haplotype diversity, and π is nucleotide diversity.

Table S5. Nucleotide diversity statistics for *Neoaulaxinia persicum* and *Pleroma menoui* at two different spatial scales

Grouping		*Neoaulaxinia persicum*								*Pleroma menoui*			
		COI				*12S*				*COI*			
		N	n	Hd	π	N	n	Hd	π	N	n	Hd	π
Region	North	2	1	0	0	1	1	-	-	7	3	0.524	0.002
	South	28	7	0.537	0.0019	10	1	0	0	9	3	0.417	0.0026
Geomorphic feature	Norfolk Ridge	-		-	-	-		-	-	4	3	0.833	0.0035
	Kermadec Ridge	7	2	0.288	0.0006	4	2	0.5	0.02547	7	2	0.286	0.0007
	Three Kings Ridge	12	3	0.318	0.0011	4	1	0	0	2	1	0	0
	NE Continental Slope	-		-	-	-		-	-	3		0.667	0.0062
	Challenger Plateau	1	1	-	-	-		-	-	-		-	-
	Hikurangi Margin	2	1	0	0	1	1	-	-	-		-	-
	Chatham Rise	8	4	0.75	0.003	2	1	0	0	-		-	-

N is the number of sequences for analysis from the site, n is the number of haplotypes detected in the examined site, n is the number of sequences for analysis from the site, Hd is haplotype diversity, and π is nucleotide diversity.

- data are unavailable.

Table S6. Microsatellite diversity statistics for *Poecillastra laminaris* at three different spatial scales

Group	Index	*ong7*	*ong26*	*ong21*	*ong39*	*ong34*	*ong27*	*ong17*	*ong48*	*ong4*	*ong8*	Mean	SE
Northern province	N	13	19	26	21	31	20	28	21	16	14	20.900	1.876
	Na	14.00	11.00	18.00	22.00	13.00	6.00	16.00	7.00	16.00	13.00	13.600	1.529
	Ne	12.071	5.270	9.869	16.036	5.754	3.433	9.333	3.600	11.378	9.333	8.608	1.285
	Ho	0.692	0.947	0.500	0.857	0.806	0.600	0.893	0.190	0.875	1.000	0.736	0.078
	He	0.917	0.810	0.899	0.938	0.826	0.709	0.893	0.722	0.912	0.893	0.852	0.026
	HWE	*	***	**	*	*	*	***	***	ns	ns		
North region	N	8	10	15	12	16	12	16	11	9	8	11.700	0.978
	Na	10.00	6.00	12.00	17.00	9.00	6.00	12.00	6.00	10.00	9.00	9.700	1.086
	Ne	9.143	2.985	8.182	14.400	3.683	3.600	8.393	4.246	6.480	7.529	6.864	1.102
	Ho	0.500	0.900	0.533	0.917	0.750	0.667	0.875	0.273	1.000	1.000	0.733	0.094
	He	0.891	0.665	0.878	0.931	0.729	0.722	0.881	0.764	0.846	0.867	0.807	0.033
	HWE	**	*	ns	ns	ns	ns	*	**	ns	ns		
Central region	N	5	9	11	9	15	8	12	10	7	6	9.200	0.940
	Na	6.00	7.00	9.00	11.00	11.00	4.00	11.00	4.00	10.00	9.00	8.200	0.879
	Ne	5.000	5.400	6.541	8.526	6.818	3.122	7.579	2.299	8.909	8.000	6.219	0.708
	Ho	1.000	1.000	0.455	0.778	0.867	0.500	0.917	0.100	0.714	1.000	0.741	0.077
	He	0.800	0.815	0.847	0.883	0.853	0.680	0.868	0.565	0.888	0.875	0.817	0.028
	HWE	ns	ns	ns	ns	*	ns	ns	**	ns	ns		
Southern province/ South region	N	6	12	16	10	21	8	15	10	7	6	11.100	1.560
	Na	8.00	10.00	16.00	12.00	14.00	8.00	7.00	6.00	10.00	8.00	9.900	1.016
	Ne	7.200	7.024	13.474	8.000	7.670	6.095	4.839	4.000	8.909	7.200	7.441	0.814
	Ho	0.333	1.000	0.500	0.700	0.810	0.875	0.933	0.200	0.571	0.500	0.642	0.084
	He	0.861	0.858	0.926	0.875	0.870	0.836	0.793	0.750	0.888	0.861	0.852	0.016
	HWE	*	ns	**	*	*	ns	ns	***	ns	ns		
Campbell Plateau	N	3	3	3	2	4	1	3	3	1	1	2.400	0.340
	Na	4.00	5.00	5.00	4.00	5.00	2.00	4.00	3.00	2.00	1.00	3.500	0.453
	Ne	3.600	4.500	4.500	4.000	4.000	2.000	3.600	3.000	2.000	1.000	3.220	0.376
	Ho	0.333	1.000	1.000	1.000	0.500	1.000	0.667	0.000	1.000	0.000	0.650	0.133
	He	0.722	0.778	0.778	0.750	0.750	0.500	0.722	0.667	0.500	0.000	0.617	0.076
	HWE	ns	ns	ns	ns	ns	ns	ns	ns	mono	mono		
Chatham Rise	N	5	9	11	9	15	8	12	10	7	6	9.200	0.940
	Na	6.00	7.00	9.00	11.00	11.00	4.00	11.00	4.00	10.00	9.00	8.200	0.879
	Ne	5.000	5.400	6.541	8.526	6.818	3.122	7.579	2.299	8.909	8.000	6.219	0.708
	Ho	1.000	1.000	0.455	0.778	0.867	0.500	0.917	0.100	0.714	1.000	0.733	0.094
	He	0.800	0.815	0.847	0.883	0.853	0.680	0.868	0.565	0.888	0.875	0.807	0.033
	HWE	ns	ns	ns	ns	*	ns	ns	**	ns	ns		
Kermadec Ridge	N	7	6	11	6	10	6	11	6	5	4	7.200	0.800
	Na	9.00	5.00	11.00	11.00	8.00	3.00	10.00	5.00	6.00	7.00	7.500	0.872
	Ne	8.167	3.273	6.914	10.286	2.985	2.880	7.563	2.571	4.167	6.400	5.521	0.854
	Ho	0.571	1.000	0.636	1.000	0.700	0.667	0.909	0.333	1.000	1.000	0.782	0.074
	He	0.878	0.694	0.855	0.903	0.665	0.653	0.868	0.611	0.760	0.844	0.773	0.035
	HWE	*	ns	ns	ns	ns	ns	ns	ns	ns	ns		
Macquarie Ridge	N	3	8	13	8	16	7	11	7	6	5	8.400	1.231
	Na	4.00	7.00	13.00	9.00	14.00	6.00	6.00	5.00	8.00	7.00	7.900	1.038
	Ne	3.600	4.741	10.903	6.095	7.111	4.900	4.321	3.769	7.200	6.250	5.889	0.691
	Ho	0.333	1.000	0.385	0.625	0.875	0.857	1.000	0.286	0.500	0.600	0.646	0.086
	He	0.722	0.789	0.908	0.836	0.859	0.796	0.769	0.735	0.861	0.840	0.812	0.019
	HWE	ns	ns	*	*	ns	ns	*	*	ns	ns		

ong7 – name of individual microsatellite locus, N=number of individuals successfully PCR amplified, Na = No. of Different Alleles, Ne = No. of Effective Alleles, Ho = Observed Heterozygosity, He= Expected Heterozygosity, ns=not significant, *=P<0.05, **=P<0.01, ***=P<0.001, mono= Monomorphic.

Table S7. *Poecillastra laminaris*

Pairwise Φ_{ST} values for *COI* (below diagonal) and *Cytb* (above diagonal) amongst populations

Populations	Kermadec Ridge	Three Kings Ridge	Challenger Plateau	NW Slope	Hikurangi Margin	Chatham Rise	Campbell Plateau	Bounty Trough	Macquarie Ridge	Antarctica
Kermadec Ridge		-	0.891**	-1.000	0.410	0.371**	0.937**	0.700*	0.400**	0.891
Three Kings Ridge	-0.875		-	-	-	-	-	-	-	-
Challenger Plateau	0.780*	1.000		1.000	0.500	-0.058	1.000*	0.421	0.432**	1.000
NW Slope	-0.875	0	1.000		-1.000	-0.063	1.000	-0.467	-0.039	1.000
Hikurangi Margin	0.264	-1.000	0	-1.000		-0.012	0.848	0.063	0.154	0.429
Chatham Rise	0.329**	-0.101	-0.104	-0.101	-0.072		0.474**	0.144	0.226**	0.309
Campbell Plateau	0.95**	1.000	1.000	1.000	0.919	0.469		0.250	0.434*	1.000
Bounty Trough	0.652*	-0.727	0.136	-0.727	0	0.148*	0.250		0.058	-0.294
Macquarie Ridge	0.650**	0.444	0.502*	0.444	0.462*	0.210**	0.467**	0.238		0.064
Antarctica	0.899	1.000	1.000	1.000	0.600	-0.092	1.000	-1.000	-0.583	

Significant values of p<0.05 are marked as *, and p<0.01 are marked as **.

- data are unavailable.

Table S8. *Poecillastra laminaris*

Pairwise F_{ST} values for microsatellite variation between populations

	Campbell Plateau	Chatham Rise	Kermadec Ridge	Macquarie Ridge
Campbell Plateau				
Chatham Rise	-0.005			
Kermadec Ridge	0.156*	0.061*		
Macquarie Ridge	0.023	-0.015	0.035	

Table S9. *Penares* sp.

Pairwise Φ_{ST} values for *COI* (below diagonal) and *12S* (above diagonal) amongst populations

Populations	Macquarie Ridge	Kermadec Ridge	Challenger Plateau	Hikurangi Margin	Chatham Rise
Macquarie Ridge		-0.263	0	0	0
Kermadec Ridge	0.029		-1.000	-1.000	0.111
Challenger	-1.000	-0.571		0	0
Hikurangi	-1.000	-0.571	0		0
Chatham	0.281	0.224	-1.000	-1.000	

Significant values of p<0.05 are marked as *, and p<0.01 are marked as **.

Table S10. *Neoaulaxinia persicum*

Pairwise Φ_{ST} values for *COI* (below diagonal) and *12S* (above diagonal) amongst populations

Populations	Kermadec Ridge	Three Kings Ridge	Chatham Rise	Challenger Plateau	Hikurangi Margin
Kermadec Ridge		0	-0.263	-	-1
Three Kings Ridge	-0.016			-	0
Chatham Rise	0.16	0.01		-	0
Challenger Plateau	0.867	0.76	0.143		-
Hikurangi Margin	0.273	0.052	-0.262	0.333	

Significant values of p<0.05 are marked as *, and p<0.01 are marked as **.

Table S11. *Pleroma menoui*

Pairwise Φ_{ST} values for *COI* amongst populations

Populations	Norfolk Ridge	Kermadec Ridge	Three Kings Ridge	NW Slope
Norfolk Ridge				
Kermadec Ridge	0.095			
Three Kings Ridge	-0.263	-0.313		
NW Slope	0.029	0.225	-0.2	

Significant values of p<0.05 are marked as *, and p<0.01 are marked as **.

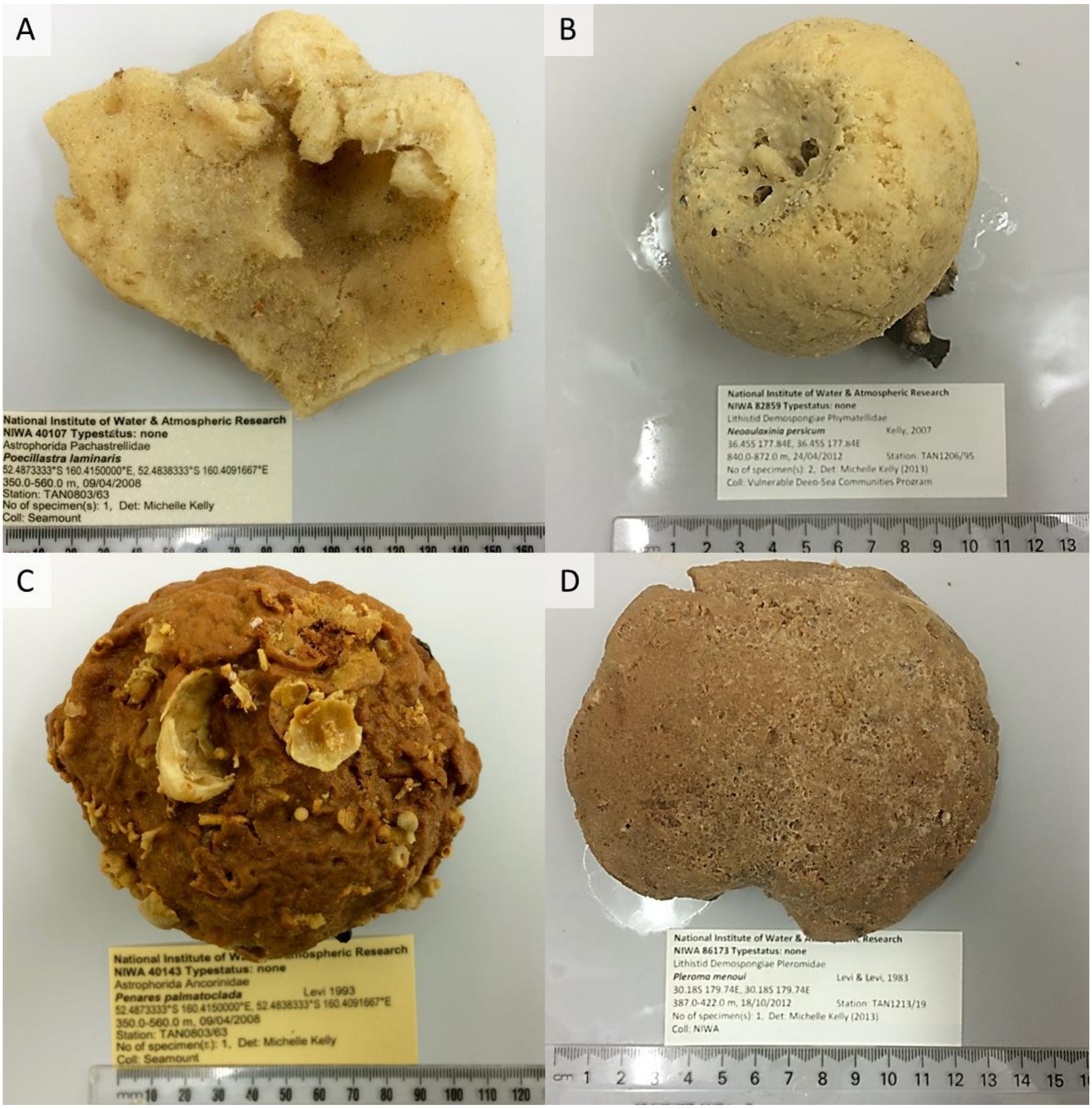

Figure S1. Exemplar specimens of (A) *Poecillastra laminaris* Sollas, 1886; (B); *Neoaulaxinia persicum* Kelly, 2007; (C) *Penares* sp.; (D) *Pleroma menoui* Lévi & Lévi, 1983.

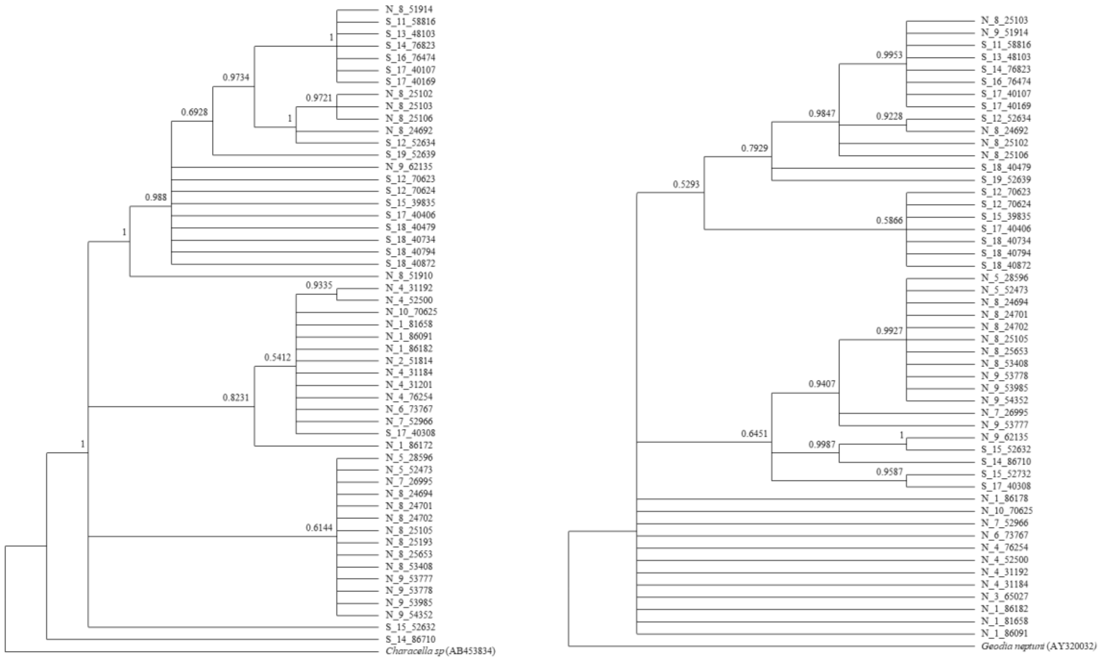

Figure S2. Bayesian phylogenetic tree of *COI* (left) and *CytB* (right) for *Poecillastra laminaris*. Letter N or S in front of sequence name indicates the Northern or Southern province, respectively.

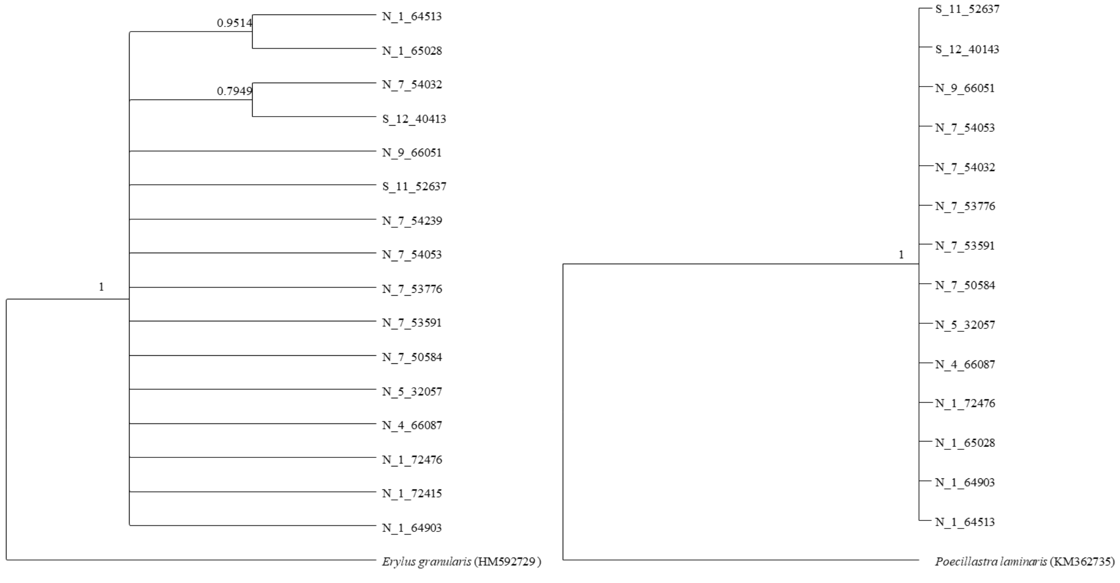

Figure S3. Bayesian phylogenetic tree of *COI* (left) and *12S* (right) for *Penares* sp. Letter N or S in front of sequence name indicates the Northern or Southern province, respectively.

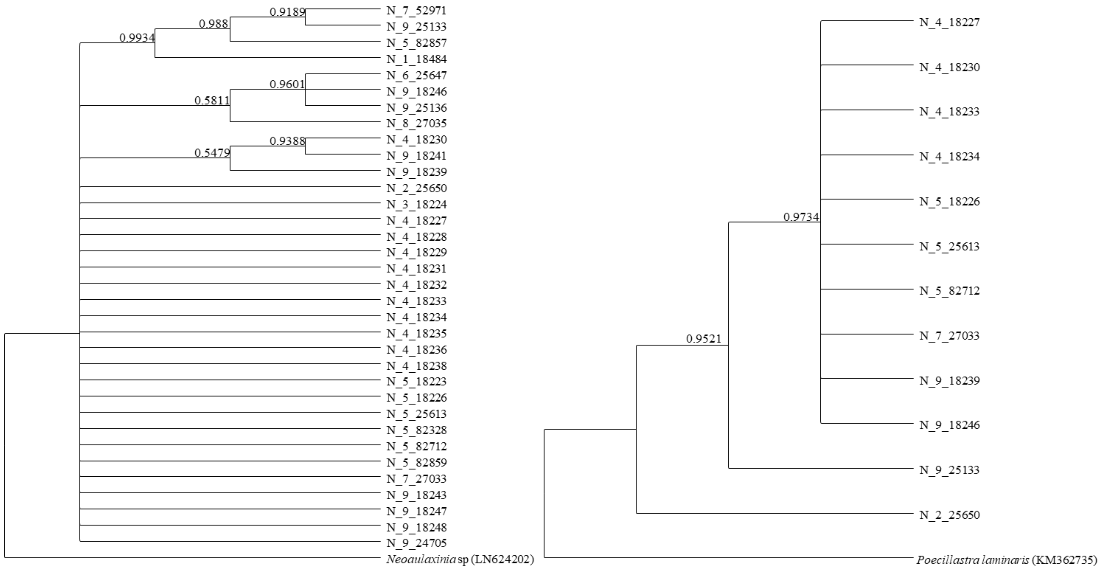

Figure S4. Bayesian phylogenetic tree of *COI* (left) and *12S* (right) for *Neoaulaxinia persicum*. Letter N or S in front of sequence name indicates the Northern or Southern province, respectively.

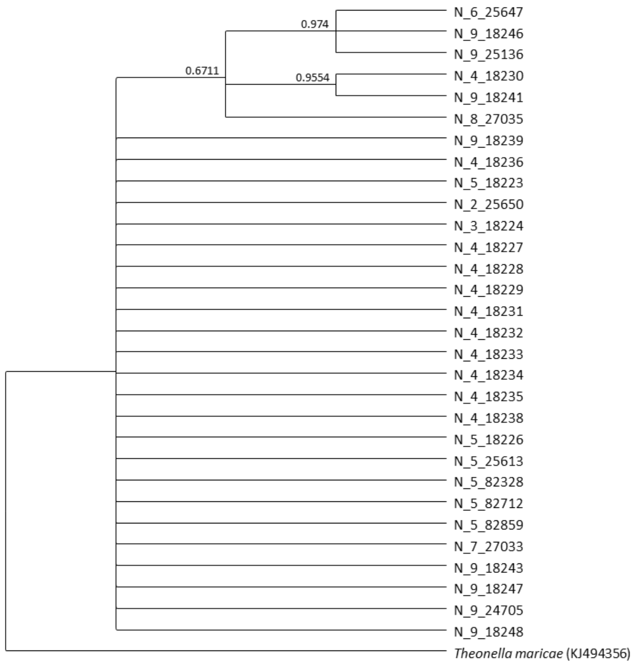

Figure S5. Bayesian phylogenetic tree of *COI* for *Pleroma menoui*. Letter N or S in front of sequence name indicates the Northern or Southern province, respectively.

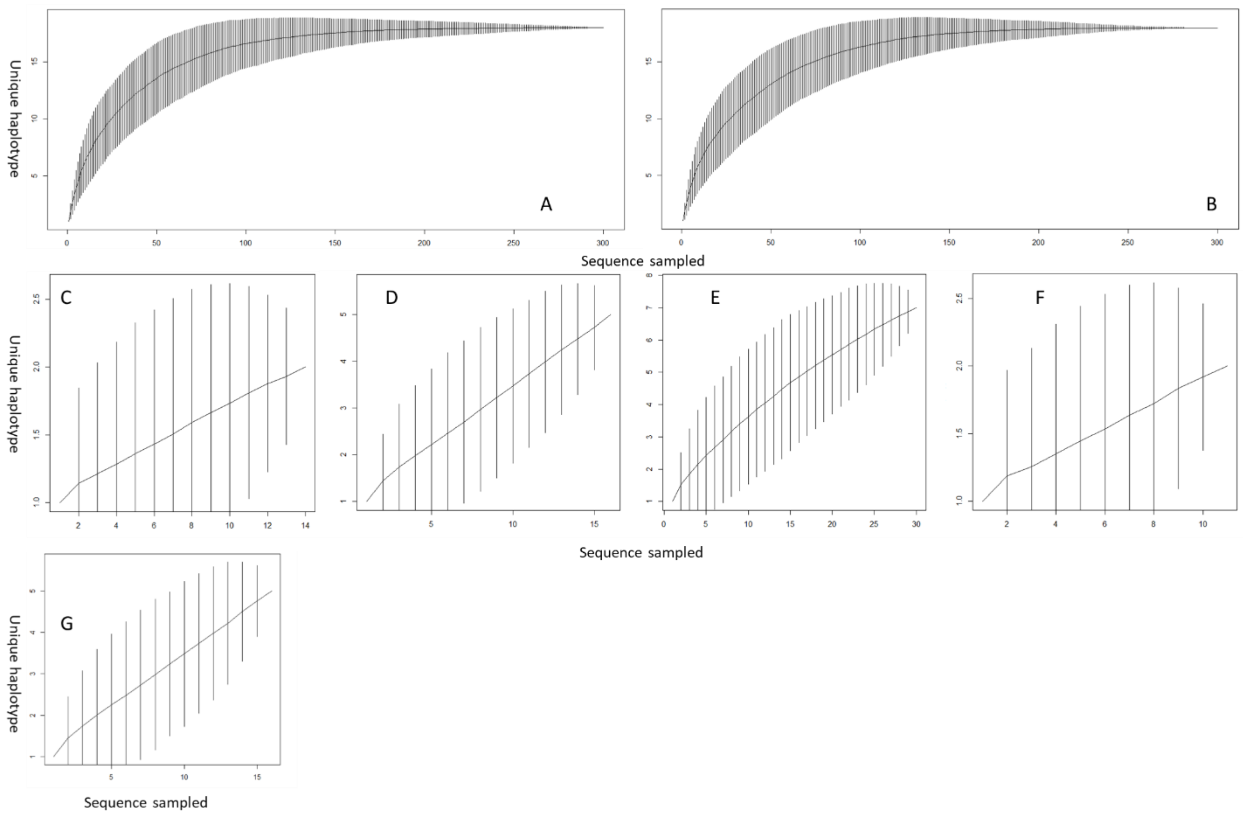

Figure S6. Sampling effect test (haplotype accumulation curves) for all species and all markers. A. *COI* of *Poecillastra laminaris*, B. *Cytb* of *Poecillastra laminaris*, C. *COI* of *Penares* sp., D. *12S* of *Penares* sp., E. *COI* of *Neoaulaxinia persicum*, F. *12S* of *Neoaulaxinia persicum*, G. *COI* of *Pleroma menoui*.

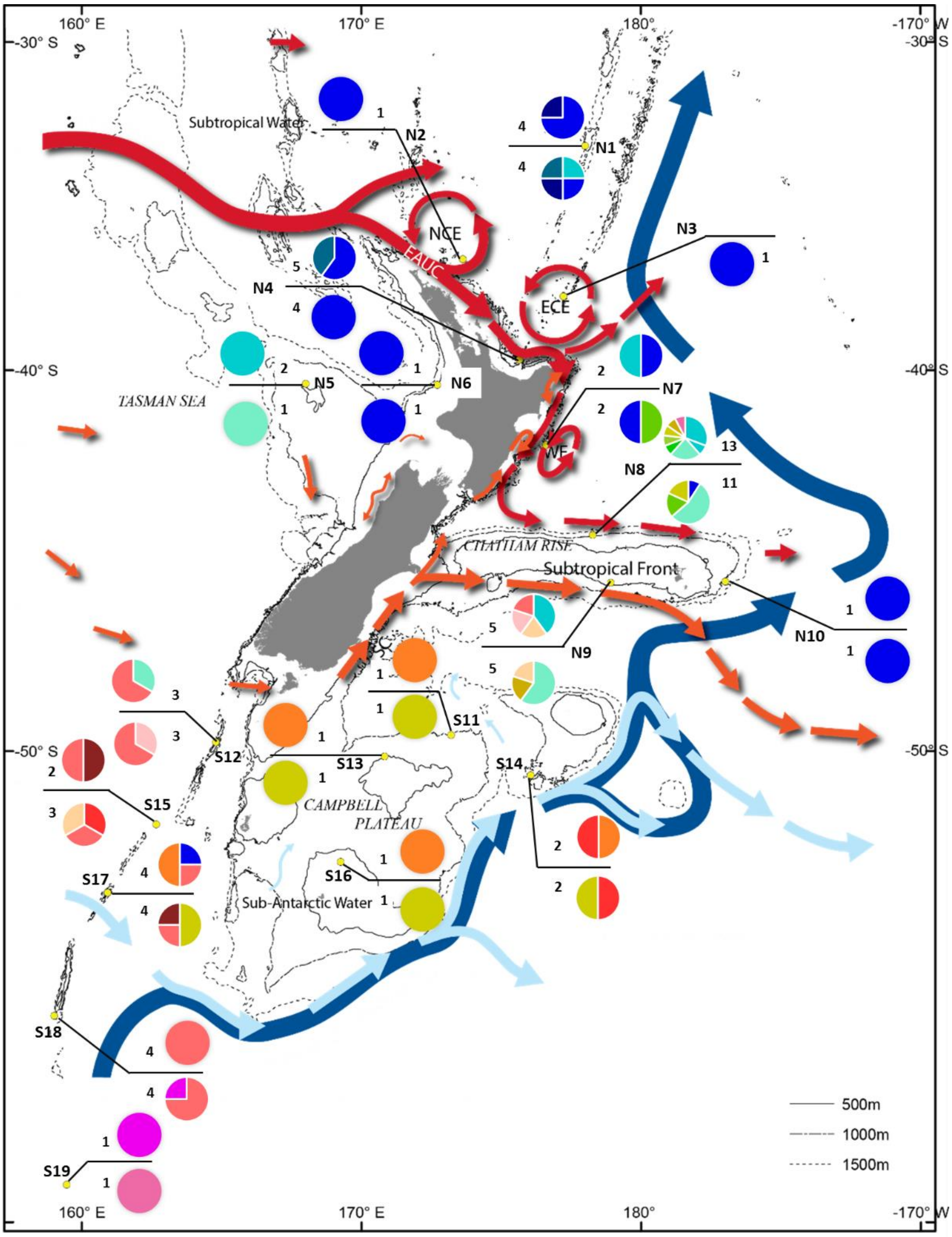

Figure S7. Haplotype map for *Poecillastra laminaris COI* (above line) and *Cytb* (below line) overlain by summary of the major currents in the New Zealand region (source Chiswell *et al*, 2015). Pie graph indicates haplotypic composition of each location and numbers indicate total number of sequences from each location. EAUC, East Auckland Currents; NCE, North Cape Eddy; ECE, East Cape Eddy; WE, Wairarapa Eddy).

Appendix S1. Sample collection acknowledgement

“Seamounts: their importance to fisheries and marine ecosystems”, undertaken by the NIWA and funded by the former New Zealand Foundation for Research, Science and Technology (FRST) with additional funding from the former Ministry of Fisheries (MFISH TAN0104,TAN0905), NOAA Satellite Operations Facility (TAN0413 cruise), and Census of Marine Life (CoML) field programme on seamounts, CenSeam (TAN0604 cruise).; Specimens collected under the Scientific Observer Program funded by the New Zealand Ministry for Primary Industries (MPI); New Zealand Cold Vents: New Vents program (RV Sonne voyage SO191) conducted by IFM-GEOMAR Leibniz-Institut für Meereswissenschaften an der Universität Kiel and partners; Fisheries research trawl surveys (TAN0501, TAN0813, TAN0901) conducted by NIWA and funded by MFish (now MPI); RENEWZ I (TAN0616) voyage, were components of the project ‘Exploration of Chemosynthetic Habitats of the New Zealand Region’, funded by NOAA Ocean Exploration and NIWA, with co-funding from Woods Hole Oceanographic Institution (WHOI), Scripps Oceanographic Institution, and the University of Hawaii; Ocean Survey 20/20 Chatham/Challenger Biodiversity and Seabed Habitat Project, jointly funded by MFish, Land Information New Zealand (LINZ), NIWA, and Department of Conservation; New Zealand-Australian “MacRidge 2” research voyage (TAN0803), the biological component of which was part of NIWA’s research project “Seamounts: their importance to fisheries and marine ecosystems” funded by FRST and CSIRO’s Division of Marine and Atmospheric Research project “Biodiversity Voyages of Discovery” funded by the CSIRO Wealth from Oceans Flagship; Kermadec Arc Minerals (KARMA, TAN1007) voyage, funded by the Ministry of Business, Innovation & Employment (MBIE), in collaboration with Auckland University, GNS Science, and WHOI; Ocean Survey 20/20 Mapping the Mineral Resources of the Kermadec Arc (voyage TAN1104), funded by LINZ, GNS, NIWA and WHOI; Biogenic Habitats on the Continental Shelf (voyages TAN1105 & TAN1108), funded by MFish, FRST, NIWA and Oceans Survey 20/20 R/V Tangaroa days funded by LINZ; ‘Impact of resource use on vulnerable deep-sea communities’ project (TAN1206), funded by MBIE; Nascent Inter-Ridge Volcanic And Neotectonic Activity (NIRVANA, TAN1213) voyage, funded by MPI, in collaboration with Auckland University, GNS, and the University of New Hampshire (USA) and with funding from the program ‘Impact of resource use on vulnerable deep-sea communities’ project, funded by MBIE; South Pacific Vulnerable Marine Ecosystems Project funded by MBIE.
